# Evaluation of Unexplained Peripheral Lymphadenopathy and Suspected Malignancy Using a Distinct Quick Diagnostic Delivery Model: Prospective Study of 372 Patients

**DOI:** 10.1097/MD.0000000000000095

**Published:** 2014-10-03

**Authors:** Xavier Bosch, Emmanuel Coloma, Carolina Donate, Lluís Colomo, Pamela Doti, Anna Jordán, Alfonso López-Soto

**Affiliations:** Department of Internal Medicine (XB, EC, CD, PD, AJ, AL-S); and Department of Pathology (Cytopathology Section) (LC), Hospital Clínic, Institut d’Investigacions Biomèdiques August Pi i Sunyer (IDIBAPS), University of Barcelona, Barcelona, Spain.

## Abstract

Although rapid diagnostic testing is essential in suspicious peripheral lymphadenopathy, delays in accessing them can be considerable. We investigated the usefulness of an internist-led outpatient quick diagnosis unit (QDU) in assessing patients with unexplained peripheral lymphadenopathy, focusing on the characteristics, diagnostic, and treatment waiting times of those with malignancy. Patients aged ≥18 years, consecutively referred from 12 primary health care centers (PHCs) or the emergency department (ED) for unexplained peripheral lymphadenopathy, were prospectively evaluated during 7 years. Diagnostic investigations were done using a predefined study protocol. Three experienced cytopathologists performed a fine-needle aspiration cytology (FNAC) systematic approach of clinically suspicious lymphadenopathy with cytomorphology and immunophenotyping analyses. We evaluated 372 patients with a mean age (SD) of 45.3 (13.8) years; 56% were women. Malignancy was diagnosed in 120 (32%) patients, including 81 lymphomas and 39 metastatic tumors. Metastatic lymphadenopathy was diagnosed by FNAC in all 39 patients and the primary tumor site was identified in 82% of them when cytomorphology and immunocytochemistry were combined. A correct diagnosis of lymphoma was reached by FNAC in 73% of patients. When accepting “suspicious of” as correct diagnosis, the FNAC diagnosis rate of lymphoma increased to 94%. Among patients with malignancy, FNAC yielded 1.3% of false negatives and no false positives. All patients with an FNAC report of correct or suspicious lymphoma underwent a surgical biopsy, as it is a mandatory requirement of the hematology department. Mean times from first QDU visit to FNAC diagnosis of malignancy were 5.4 days in metastatic lymphadenopathy and 7.5 days in lymphoma. Mean times from receiving the initial referral report to first treatment were 29.2 days in metastatic lymphadenopathy and 40 days in lymphoma. In conclusion, a distinct internal medicine QDU allows an expeditious, agile, and prearranged system to diagnose malignant peripheral lymphadenopathy. Because of the close collaboration with the cytopathology unit and the FNAC methodical approach, diagnostic and treatment waiting times of patients with malignancy fulfilled national and international time frame standards. This particular diagnostic delivery unit could help overcome the difficulties facing PHC, ED, and other physicians when trying to provide rapid access to investigations to patients with troublesome lymphadenopathy.

## INTRODUCTION

Peripheral lymphadenopathy has a wide differential diagnosis and causes concern among patients and doctors alike due to the possibility of a missed or delayed diagnosis of malignancy.^1^ Compared with the general population, patients with enlarged lymph nodes were recently reported to have a significantly increased risk of hematological and solid cancer during follow-up.^2^ Therefore, especially in patients with troublesome peripheral lymphadenopathy, quick diagnostic testing can be essential.

In countries such as Spain, where 90% of hospitals are public, diagnostic tests such as CT scans and endoscopies may take several weeks or even months when ordered by primary health care center (PHC) physicians. Furthermore, patients requiring cytological or histological studies can only be assessed in the hospital setting, unless the patient has a private provider. However, although most patients with peripheral lymphadenopathy are referred to the outpatient clinics of the reference hospital to speed up diagnostic procedures,^3–5^ even in this setting, some diagnostic examinations may take several weeks. In some cases, PHC physicians refer patients directly to the emergency department (ED), hoping to gain faster access to diagnostic investigations.^6,7^

Outpatient quick diagnosis units (QDUs), based in hospitals and headed by internists, are a proposed solution for delays in the diagnosis of patients with suspected serious disease, most notably cancer, decreasing PHC referrals to ED and helping avoid hospitalizations for diagnostic workup. Although still little known today, these types of units have been tested and exist in Europe, and they have been essentially studied in Spain.^6–9^ Because of their dynamic, agile functioning and the savings generated, QDUs are viewed as having strong implications in Spain and possibly other public funded health systems such as the UK, where outpatient practice is also under intense pressure.^7–9^ Exploring the potential implications of implementing QDU in the United States, US investigators recently argued that because most primary care physicians in this country are unlikely to provide regular and frequent access to unscheduled care, these units could provide an innovative healthcare diagnostic service with the potential to attain the objective of cost-effective high quality for everyone.^9^

Patients with new onset unexplained peripheral lymphadenopathy, including those with subsequent confirmation of malignancy, may have few or no associated general symptoms,^1,10–12^ and are therefore especially suitable for QDU evaluation. However, no study has assessed the significance of this model for the diagnosis and management of this condition.

Encouraged by our initial fruitful collaboration with the cytopathology unit of our hospital, we decided to investigate the convenience and usefulness of a QDU of a tertiary, university hospital in Barcelona in evaluating patients referred for unexplained peripheral lymphadenopathy, focusing on the characteristics, diagnostic, and treatment waiting times of those with metastatic lymphadenopathy and hematological malignancies.

## MATERIALS AND METHODS

### The Unit

Patients with potentially serious disease who are well enough to travel to the QDU and the hospital diagnostic services on an outpatient basis are evaluated at the QDU, sited in an 870-bed third level hospital in Barcelona (Spain) with a reference population of almost 550,000. Most referrals come from the hospital ED and a dozen PHC. The characteristics, functioning, and referral criteria of the unit have been reported elsewhere.^6,7^

### Study Design and Population

We prospectively evaluated patients aged ≥18 years, who were consecutively referred to the QDU from PHC or ED between July 2006 and September 2013 because of unexplained peripheral lymphadenopathy. Inclusion criteria were peripheral lymphadenopathy detected by the referring physician on physical examination or imaging studies (eg, ultrasonography) and reported as the main reason for referral to QDU, and no apparent diagnosis after at least 1 visit at the PHC or ED. Patients in whom lymphadenopathy resolved or was not identified at the first QDU visit and patients lost to follow-up were excluded. The study was approved by the Hospital Clínic research ethics committee. Informed consent was obtained from all patients included.

### Diagnostic Protocol of Unexplained Peripheral Lymphadenopathy

Each patient was evaluated by the attending physician of the QDU, a consultant internist, who did a complete anamnesis and physical examination and initiated a diagnostic study of unexplained peripheral lymphadenopathy according to a specific protocol. To objectively assess the size of the enlarged lymph node and monitor any growth or reduction, a plastic caliper was used to measure its surface palpability (long and short axes). When there were clinical doubts about the significance of a lymph node, an ultrasonography of the region was carried out.

Different protocol-driven diagnostic tests were performed according to clinical presentation and characteristics of lymphadenopathy. Laboratory tests included, among others, acute phase reactants (C-reactive protein and erythrosedimentation rate), hemogram (total leukocytes, manual white blood cell count, hemoglobin, hematocrit, and platelets), liver function tests, serum lactate dehydrogenase, serum total proteins and protein electrophoresis, microbiologic serologies (eg, IgM and IgG for cytomegalovirus, Epstein–Barr virus, *Toxoplasma gondii*, and human parvovirus B19), human immunodeficiency virus testing, serum β2 microglobulin, and serum tumor markers including carcinoembryonic antigen, prostate-specific antigen (men), cancer antigen 15-3 (women), cancer antigen 19-9, neuron-specific enolase, and cancer antigen 125.

During successive visits, results of initial diagnostic studies were checked over and the course of disease was evaluated. As predefined by the study protocol, when a diagnosis had not been reached, additional investigations were performed as appropriate in accordance with the results of the previous ones and the clinical course of disease.

#### Cytological and Histological Investigations

Fine-needle aspiration cytology (FNAC) was carried out according to the characteristics, size, and site of lymphadenopathy. In general, regardless of size, any patient with hard and fixed lymphadenopathy underwent an FNAC. When various lymph nodes were palpable, the largest, most suspicious, and accessible was chosen.

The FNAC procedure was performed on 2 days a week by a team of 3 cytopathologists in a specific cytology room. The cytopathologist who performed the FNAC also examined the samples. The procedure was performed with 23-G or 25-G needles and the quality of the sample obtained was evaluated on-site. One-half to two-thirds of the slides obtained in the first needle pass were quickly stained (Panoptic; Grifols, Barcelona, Spain) and immediately examined. When infection was suspected, staining, cultures, and polymerase chain reaction on FNAC specimens were performed. When the smear favored the diagnosis of epithelial metastasis, 1 or 2 additional passes were repeated to obtain material for the cellblock preparation for immunocytochemical studies. If lymphoma was suspected after the on-site evaluation, further passes were repeated to obtain material for flow cytometry analysis. Cytology and flow cytometry samples were labeled as insufficient if the material was scarce or autolytic. Flow cytometry and immunocytochemical studies were done in the hospital immunophenotyping unit. Patients with a correct or suspicious diagnosis of lymphoma by FNAC underwent a surgical biopsy (usually at a later stage), as it is a requirement of the hematology department.

For difficult-to-access lymphadenopathy, FNAC was ultrasound guided by a radiologist and the material was also assessed by a cytopathologist.

### Onward Referrals and Follow-Up

Patients with obvious or suspicious lymphoma by FNAC were immediately referred to the hematology department, whether or not the mandatory biopsy had already been performed. Staging of lymphoma was not systematically done. Patients with solid malignancies were referred to the oncology department upon receipt of the FNAC report. Patients with reactive lymphadenopathy by FNAC and those with undiagnosed lymphadenopathy in whom FNAC was considered unnecessary were reassured and discharged from the QDU. These patients were reassessed at 2, 4, and 6 months after discharge to check any change in lymphadenopathy.

### Database

Details on patients’ demographic and epidemiological data, past history of malignancies, previous administration of medications, clinical presentation and course of disease, associated signs and symptoms, and laboratory, imaging, endoscopic, FNAC, and biopsy results were prospectively registered in a database. We also tabulated the source of referral; number and date of QDU visits; waiting times between visits; time to diagnosis; type, number, and date of diagnostic tests; final diagnosis; onward referral; and follow-up. For lymphadenopathy, we recorded the presence of local symptoms, duration from onset, size (≥1 cm or <1 cm), characteristics (soft, mobile, well demarcated, tender, hard, and fixed), number [localized (only 1 anatomic region of nodal drainage involved) or generalized (≥2 anatomic regions)], and site.

### Statistical Analysis

A descriptive analysis was made. We used stepwise logistic regression to determine the risk of malignancy in enlarged lymph nodes, including the following independent variables: age, sex, ethnicity, fever, weight loss, night sweats, duration, characteristics, size, site, and number of lymphadenopathies. The risk ratios (RRs) with 95% confidence intervals (CIs) for the various factors were calculated. A *P* value <0.05 was considered statistically significant. Data analysis was made using the SAS v.9.1 statistical package (SAS Institute Inc, Cary, NC).

## RESULTS

A total of 394 patients were referred to the QDU during the study period. Their mean age (SD) was 44.9 (13.1) years and 220 (56%) were women. Twenty-two patients were excluded, 6 were lost to follow-up after the first visit, and 16 had no palpable lymphadenopathy on the first visit.

### General Characteristics of the Study Population

We evaluated 372 patients, mostly white, with a mean age of 45.3 years (range, 18–92 years) and 207 (56%) women. Five patients had a history of malignancy. Patients were referred from PHC (64%) and ED (36%). The mean time from receiving the referral report to the first QDU visit was longer in patients referred from PHC than ED (Table 1). The mean number of QDU visits per patient was 3.4 (1.2) (median [P25;P75] 2.9 [2.6;3.2]). Table 2 shows the accompanying signs and symptoms on presentation and Table 3 the diagnostic examinations performed.

**TABLE 1:**
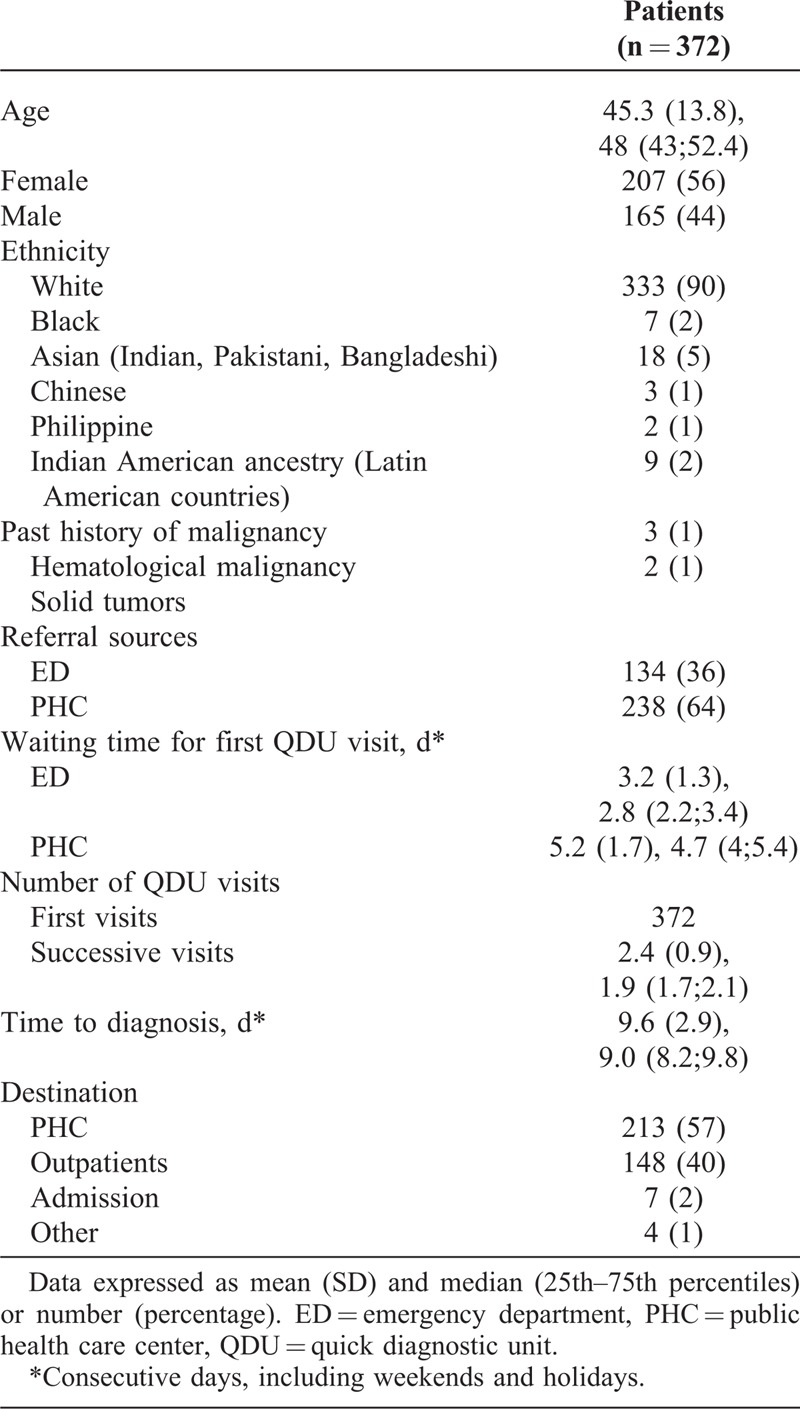
Main Characteristics of Study Patients

**TABLE 2:**
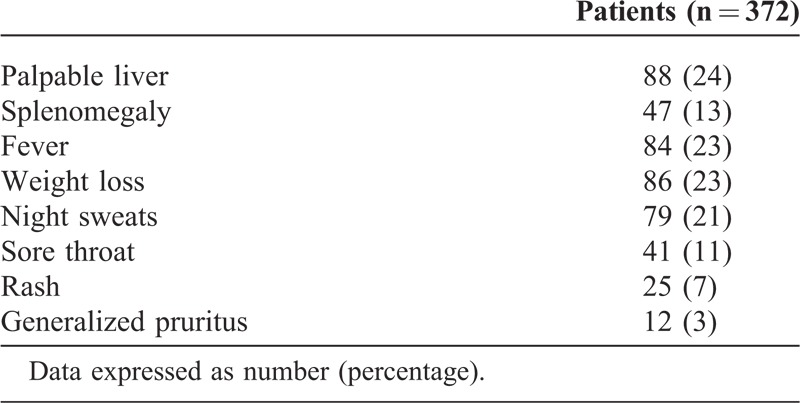
Accompanying Signs and Symptoms on Presentation

**TABLE 3:**
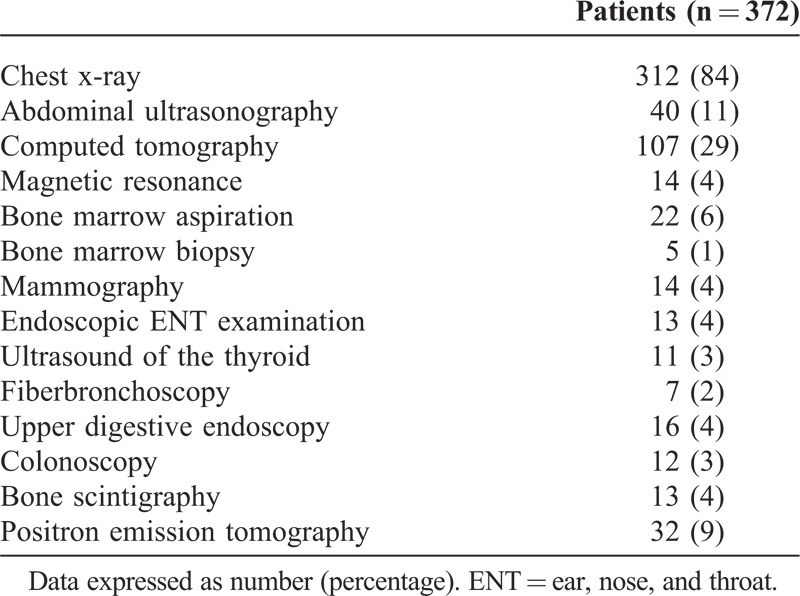
Main Diagnostic Investigations

### Final Diagnoses

The most frequent diagnosis was reactive lymphadenopathy as diagnosed by FNAC in 110 (30%) patients (mean age 38 [10.3] years; median 39.8 [37.1;43] years). Ninety-one (24%) patients had infections, including viral infections in 45 (12%) patients (mean age, 32 [9.2] years; median, 34.1[31;36.5] years) and tuberculosis lymphadenitis in 26 (7%) patients (mean age, 34.1 [10.6] years; median, 36 [34.2;39.1] years). A marked lymphocytosis (>50% of leukocytes) with atypical cells comprising >10% of leukocytes was observed in 40% of patients with viral infections and 38% of those with toxoplasmosis. Malignancy was diagnosed in 120 (32%) patients, including 81 lymphomas and 39 metastatic tumors (Table 4). The mean age of patients with metastatic tumors was 62.6 (16.1) years (median, 64.9 [60.1;68] years). Tables 4 and 5 list the specific diagnoses and the characteristics of lymphadenopathy in the main diagnostic groups, respectively.

**TABLE 4:**
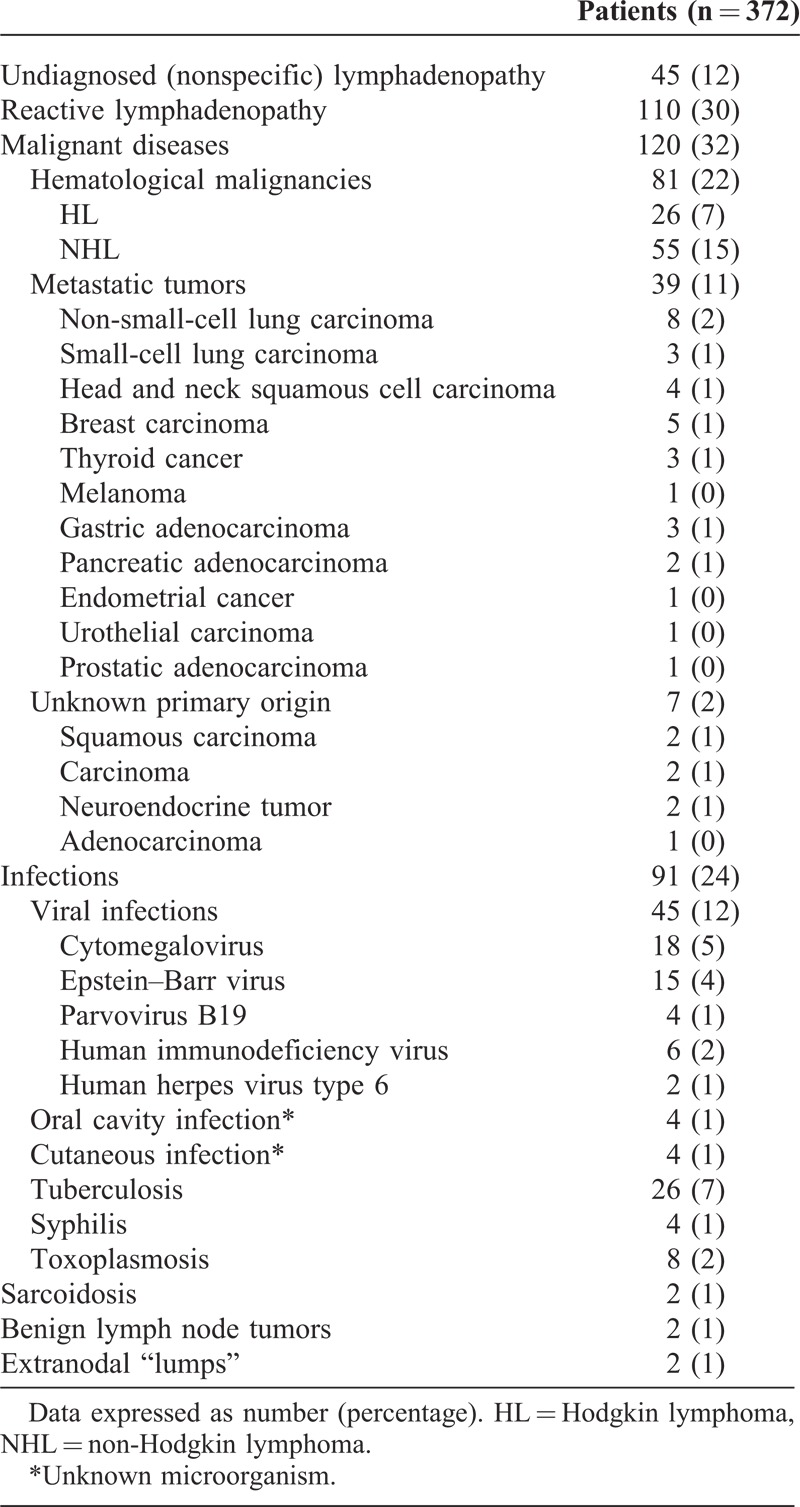
Final Diagnoses of Study Patients

**TABLE 5:**
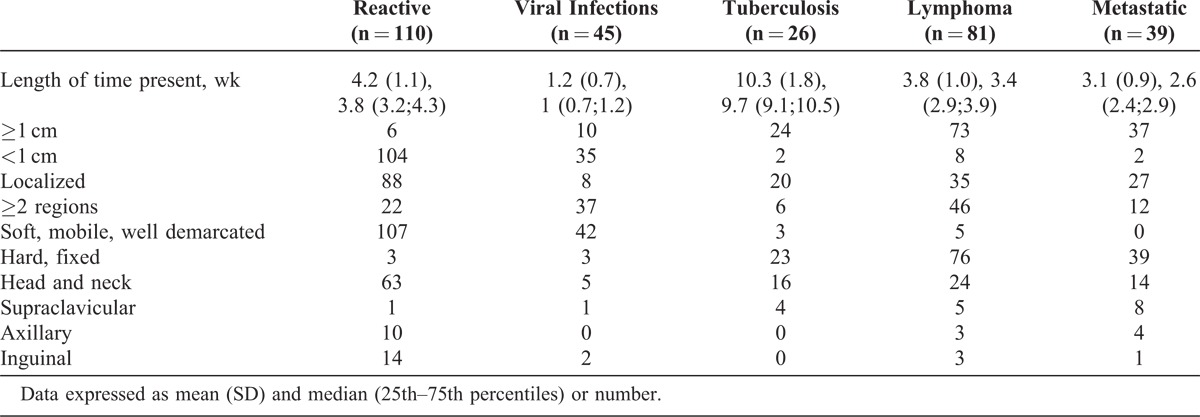
Characteristics of Lymphadenopathy in Main Diagnostic Groups

#### Cytological and Histological Results

Overall, FNAC was performed in 268 (72%) patients and biopsy in 81 (22%) patients. Based on the characteristics of lymphadenopathy and clinical manifestations, 104 (28%) patients did not undergo FNAC or biopsy.

Reactive lymphadenopathy was diagnosed by FNAC in 110 patients: 92 by both cytomorphology and flow cytometry, 14 by flow cytometry (suspicious cytomorphology findings in 7 and insufficient material for cytomorphology in 7), and 4 by cytomorphology (insufficient material for flow cytometry in all 4).

Metastatic lymphadenopathy was diagnosed by FNAC in 39 patients, which included 5 suspicious cases. In 37/39 (95%) patients with metastatic lymphadenopathy, FNAC was ordered at the first QDU visit. Material was sufficient for evaluation of cytomorphology or cytomorphology and immunocytochemistry in all patients. Combining both approaches, the primary tumor site was identified in 32 (82%) patients, which included 4 suspicious cases (2 non-small-cell lung carcinoma, 1 small-cell lung carcinoma, and 1 gastric adenocarcinoma) (Table 4).

Cases of non-Hodgkin lymphoma (NHL) (n = 55) and Hodgkin lymphoma (HL) (n = 26) were fully subtyped by biopsy. In 78/81 (96%) patients with lymphoma, FNAC was ordered at the first QDU visit. Among all 81 patients with lymphoma, 17 (21%) had a suspicious diagnosis and 1 had reactive changes by FNAC. Material was insufficient for cytomorphology in 5% of cases (all HL) and for flow cytometry in 21% (12% NHL and 9% HL). By combining cytomorphology and flow cytometry results, a correct diagnosis of lymphoma was made in 73% of cases. When accepting “suspicious of” as correct diagnosis, the diagnosis rate increased to 94% (Table 6). The different subtypes of NHL according to cytomorphology and flow cytometry findings are shown in Table 6. Fifty-four percent of patients with HL were correctly diagnosed by FNAC. Histopathological diagnoses of HL included 15 cases of nodular sclerosis classical HL, 6 of mixed cellularity classical HL, 3 of lymphocyte-rich classical HL, and 2 of nodular lymphocyte predominant HL.

**TABLE 6:**
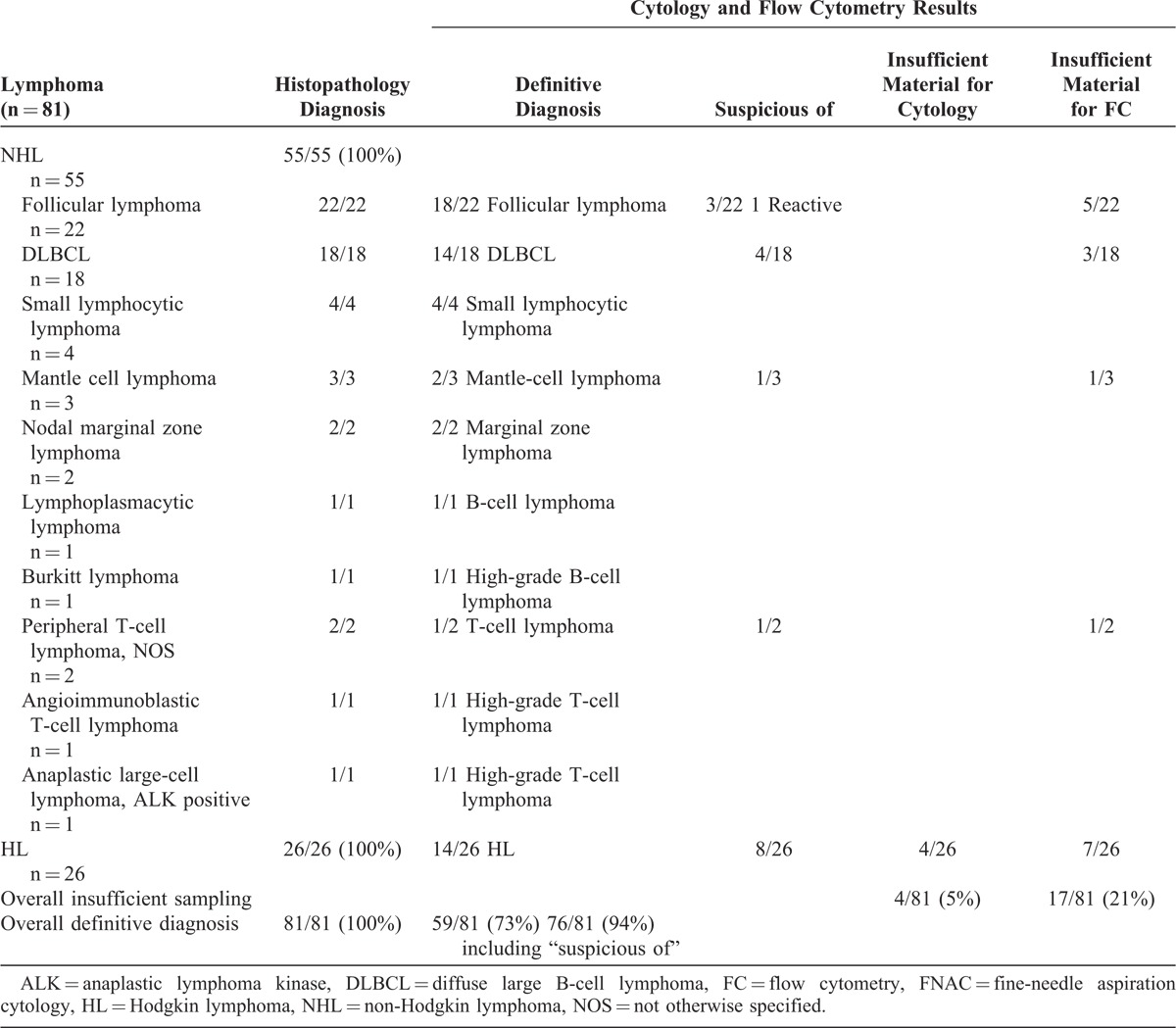
Histopathological, Cytological, and Flow Cytometry Results in Patients With Lymphoma, Sampled by Surgical Biopsy and FNAC

### Waiting Times in Patients With Malignancy

In patients with malignancy, the mean wait from receiving the initial referral report to the first QDU visit was 2.9 (1.1) days (median, 2.5 [1.9;3.2] days) in patients referred from ED and 4.8 (1.6) days (median, 4.4 [3.6;5.2] days) in those referred from PHC. In addition, the mean time from the first visit to the FNAC procedure was 2.1 (1.0) days (median, 1.8 [1.5;2.0] days).

Regarding diagnosis, the mean time from the first visit to the FNAC diagnosis was 5.4 (1.8) days (median, 4.9 [4.1;5.8] days) in metastatic lymphadenopathy. In lymphomas, the mean time between the first visit and the FNAC or biopsy (when FNAC failed because of false negatives [ie, reactive changes in 1 case] or insufficient material for cytomorphology evaluation [ie, 4 HD cases]) diagnosis was 7.5 (2.5) days (median, 7 [6.0;8.1] days).

Following diagnosis and QDU discharge, the mean wait for an appointment of an oncologist and a hematologist was 4.2 (1.6) days [median, 3.8 (3.3;4.2) days] and 3.3 (1.2) days (median, 2.8 [2.3;3.4] days), respectively.

As mentioned, all patients with lymphoma underwent a formal mandatory biopsy regardless of the FNAC diagnosis. This biopsy was immediately ordered by the QDU attending physician upon receipt of the FNAC diagnostic report and, in order to save time, mostly performed after QDU discharge. Specifically, the mean time between an FNAC report of correct or suspicious lymphoma and the biopsy procedure in all 76 patients with this diagnosis was 6.1 (2.2) days (median, 5.6 [4.8;6.3] days), whereas the mean time between the biopsy procedure and its diagnostic report was 5.7 (2.0) days (median, 5.2 [4.8;5.6] days).

With regard to treatment, the mean time from the FNAC (and biopsy in 5 lymphoma patients) report to the first treatment (chemotherapy or radiotherapy) was 28.3 (3.7) days (median, 27.2 [25.8;28.6] days) in patients with lymphoma and 19.6 (3.1) days (median, 18.7 [17.6;19.8] days) in those with metastatic lymphadenopathy.

Overall, the full mean time from receiving the initial referral report to the first treatment was 40 (3.8) days (median, 39.1 [37.5;40.7] days) in patients with lymphoma and 29.2 (3.4) days (median, 28.0 [26.7;29.3] days) in patients with metastatic lymphadenopathy.

### Follow-Up of Patients With Nonmalignant Lymphadenopathy

At 6 months of follow-up, lymphadenopathy was palpable in only 8% of patients with reactive lymphadenopathy and in 9% with undiagnosed lymphadenopathy who did not undergo FNAC. Palpable lymph nodes were all <1 cm and all patients were symptom free.

### Multivariate Analysis

Five independent factors were associated with malignant lymphadenopathy: increasing age (RR = 1.31; 95% CI, 1.05–1.67; *P* = 0.02), male sex (RR = 2.53; 95% CI, 1.54–3.93; *P* = 0.01), size ≥1 cm (RR = 4.43; 95% CI, 2.77–6.48; *P* < 0.001), supraclavicular region (RR = 4.72; 95% CI, 3.02–6.79; *P* < 0.001), and hard and fixed characteristics (RR = 15.36; 95% CI, 13.27–17.61; *P* < 0.001).

## DISCUSSION

Although the incidence of peripheral lymphadenopathy in the general population is low and estimated at 0.6% to 0.7%, the prevalence of malignancy depends on the health care setting and patients’ age, ranging from 0.4% in patients aged <40 years to 4% in those aged >40 years in primary care.^1,10–12^

In our QDU, purposely designed to evaluate patients with potentially serious diseases, 32% of those referred for unexplained peripheral lymphadenopathy had malignancy (lymphomas in 68% and metastatic tumors in 33%), mostly diagnosed by a first quick FNAC approach. In a 2003 study by Chau et al^10^ of a UK rapid access lymph node diagnostic clinic, to which patients were referred from PHC, the prevalence of malignancy was 17% (95/550 patients) (lymphomas in 65% and metastatic tumors in 31%). Although the general functioning of this unit was somewhat similar to ours, their patients were initially assessed by medical oncologists,^10^ whereas QDU patients were assessed by internists. A distinctive feature of our QDU (and most Spanish internal medicine QDU) is that it is not “monothematic” but several unrelated conditions, all of them potentially associated with cancer, are evaluated including, among others, enlarged lymph nodes, unintentional weight loss, palpable abdominal masses, lung and/or pleural abnormalities suggestive of neoplasm, severe anemia, rectorrhagia, and unexplained fever.^6–8^

The concept of quick and early diagnosis (QED) was pioneered by Kendall et al^13^ in 1996 in *The Lancet*. These authors described the suitability of a hospital QED unit in the UK, mainly for patients with suspected malignancy referred from PHC. Patients underwent prompt diagnostic workup without admission and were evaluated by different consultants according to the reason for referral (eg, urologists for hematuria or testicular swellings, endoscopists for gastrointestinal bleeding, or breast surgeons for breast lumps).^13^ A more elaborated conception was defined by our group, and named the “quick diagnosis unit.”^14^ Although we have reported that QDU is a cost-saving and efficient alternative to conventional hospitalization for diagnostic purposes,^6–8^ cancer represents the most common diagnosis in Spanish QDU (18%–30% of cases).^9^

Instances of international and national proposals and policies to speed up the diagnosis of cancer from suspicion abound.^15,16^ Shortening the times between suspected and confirmed diagnosis is a cancer health care priority of the World Health Organization.^17^ The UK government in 2000 set up the National Health Service (NHS) Cancer Plan, establishing waiting time standard targets. For instance, PHC physicians should “urgently” refer any patient with suspected malignancy, including those with lymphadenopathy >1 cm persisting for 6 weeks, to a hospital specialist within 2 weeks for first assessment.^18–20^ Although far less ambitious and elaborate than the UK NHS Cancer Plan, the 2006 (updated in 2010) Spanish Health System Cancer Strategy advocates a first confirmatory test within 2 weeks of suspected malignancy.^21^ In our study, the mean wait from receiving the initial referral report to the first QDU visit (where important diagnostic decisions were made [eg, FNAC in ≥95% of patients in about 2 days]) was short: nearly 3 days for ED referrals and 5 days for PHC referrals. This wait was longer in patients referred from PHC because these referrals require preappointment checking by the QDU physician, whereas ED referrals do not.^6^

The Spanish Cancer Strategy also promoted the establishment of circuits providing rapid access to diagnostic resources to patients with well-founded suspicion of cancer.^21^ The UK Department of Health also realized that the most important primary care priority in improving early diagnosis was improved access to diagnostic tests,^20^ leading to a 2007 revision of the NHS Cancer Plan with nationwide implementation of multidisciplinary teams including one-stop clinics offering quick access in district hospitals.^22^ Undoubtedly, PHC physicians play an essential role in the initial management of cancer suspicion and diagnosis.^3,8,23,24^ Although unreported, the QDU model could successfully overcome the barriers to rapid diagnosis in patients with unexplained lymphadenopathy, at least in public health systems like Spain, where there is often a poor coordination between primary and hospital care, meaning that, in practice, only inpatients are prioritized for quick diagnostic testing.^6–8,14^

Malignancy was promptly diagnosed. The longer mean time to diagnosis in patients with lymphoma compared with metastatic lymphadenopathy (7.5 vs 5.4 days, respectively) is likely explained by the uselessness of FNAC in 6% of the former patients, who required a biopsy at a later stage. Overall, these results compare favorably with studies revealing that time to diagnosis in lymphoma can be inappropriately lengthy.^16,25–28^ Furthermore, our mean waits from diagnosis to first treatment (about 28 days in lymphoma and 20 days in metastatic lymphadenopathy) and from receiving the PHC referral report to first treatment (40 days in lymphoma and approximately 29 days in metastatic lymphadenopathy) amply complied with UK NHS treatment waiting time standards (31 days from diagnosis/decision to treat to first treatment and 62 days from urgent PHC physician referral to first treatment)^18–20^ and also with Spanish Health Ministry targets (30 days from diagnosis to first treatment).^29^ Treatment waiting times were longer in patients with lymphoma as a result of the additional surgical biopsy performed on all of them.

Is there an advantage of QDU over conventional diagnostic pathways regarding waiting times in lymphoma and metastatic lymphadenopathy? Before answering this question, some considerations should be made. Comparing results of cancer waiting times is usually problematic due to different methods of data gathering (eg, medical records and surveys), different values (eg, median or mean), and different time periods.^25,28^ Despite the efforts of cancer registries to define and standardize the variables they gather, there is no general consensus on the indicators and methods to establish treatment waiting times.^4,30^ Furthermore, in numerous countries, including Spain, public health bodies do not systematically monitor cancer time periods, and studies have largely been carried out in hospitals^4,31^ or relied on data from primary care registries.^26,28^

Research studies on waiting times in lymphoma are relatively scarce. Unlike other malignances, the path to diagnosis can be especially difficult. In the absence of peripheral lymphadenopathy, clinical presentation may be imprecise and broad and symptoms may also be seen in patients with benign disease, purportedly resulting in protracted diagnostic and treatment waiting times.^25,27^ A UK National Institute for Health and Care Excellence guideline acknowledges that delayed diagnosis in lymphoma may be a consequence of wrong referral pathways from PHC.^32,33^

After performing a literature review, our predefined diagnostic waiting times in lymphoma were shorter than those reported in 3 research studies.^10,26,27^ In the aforementioned study by Chau et al^10^ of a quick lymph node diagnostic clinic, the median time from the first clinic visit to diagnosis was 21 days in HL and 24 days in diffuse large B-cell lymphoma (DLBCL) (median of 7 days in all lymphoma types in our study). Two studies using a conventional pathway (ie, PHC referral to hospital) revealed a median time from referral to diagnosis of 55 days in all lymphoma types^27^ (median of 11.2 days in our study) and a mean time from first hospital visit to diagnosis of 14.5 days in NHL^26^ (mean of 7.5 days in all lymphoma types in our study), respectively. With regard to treatment waiting times, in the study by Chau et al,^10^ the median time from receiving initial referral report to the first treatment was 35 days in HD and 39 days in DLBCL (median of 39.1 days in all lymphoma types in our study). No other studies have examined the treatment waiting times in lymphoma using our predefined times. Although prehospital delays (eg, patient and PHC delays) were not analyzed in our study, an article published by Danish researchers^16^ revealed a median time from first PHC physician investigation to first treatment of 60 days in NHL.

Unlike lymphoma, no previous studies have explicitly reported the waiting times in metastatic lymphadenopathy as we did. Nevertheless, some authors have described the specific times in local and disseminated disease among patients hospitalized for diagnosis and treatment of solid cancer. A Spanish study of 1023 patients diagnosed with the 6 most incident cancers in 22 hospitals found shorter times from diagnosis to first treatment in disseminated than in local stage, with median times varying according to the cancer site. For instance, in disseminated disease, it was 17.5 days in breast cancer, 20 days in colorectal cancer, and 24 days in lung cancer.^31^ Our median time from diagnosis to first treatment in metastatic lymphadenopathy was 18.7 days. In another Spanish study of 7223 patients diagnosed with different solid malignancies in a public hospital in Barcelona, median times from diagnosis to first treatment also varied depending on tumor location (eg, 12 days in stage IV lung cancer, 14 days in stage IV colorectal cancer, and 25 and 18 days in stage III and IV breast cancer, respectively).^4^ Of concern, a recent report from the Spanish Health Ministry revealed that, during 2009, the mean time from diagnosis to first treatment surpassed 30 days (the standard target) in 44% of breast cancer patients (any stage other than in situ) from 93 hospitals and in 55% of colorectal cancer patients (any stage other than in situ) from 97 hospitals.^29^

Undeniably, the collaboration between the QDU and the cytopathology unit, with a first clinical selection of candidates to FNAC by the QDU internist and the cytopathologists’ systematic approach, was essential to achieving these waiting times. As it is well known, FNAC is a quick, economical, and minimally invasive method for the diagnosis of a range of benign and malignant epithelial processes and is also used to assess lymphoma recurrence.^34–36^ The reported accuracy of FNAC for diagnosing metastatic lymphadenopathy may be >90% using only conventional morphology,^10,36^ and, when combined with immunocytochemistry, the tumor site may be identified in a significant proportion of cases, up to 86% in a recent study,^37^ in line with our results.

Regarding lymphoma, although its primary diagnosis and classification is conventionally made by biopsy, in the last decade and a half, several studies have reported that FNAC can also have a relatively high diagnostic sensitivity and specificity, mainly in NHL, when using the cytomorphology/immunophenotyping combination.^34,36–43^ In some instances, the reported diagnostic rates of this combination (assisted sometimes by cytogenetic and/or molecular studies) have led authors to claim that FNAC may not be necessarily accompanied by histopathology, whereas others have stated that NHL may be accurately subtyped.^34,36,39,40,42,43^ However, diagnosis of lymphoma by FNAC remains controversial and there is a consensus among oncologists and hematopathologists that histopathology is essential to drive therapeutic decisions.^40,44,45^ Not only some lymphomas are difficult to evaluate using FNAC (eg, HL [particularly lymphocyte-depleted classical HL, due to the scarcity of Reed–Sternberg cells and the mixture of nonmalignant cells], T-cell-rich B-cell lymphoma, and T-cell NHL^45^] but also diagnostic limitations include insufficient material to perform flow cytometry or additional stains, grading, sampling error, and, in general, loss of architecture.^37,40,45,46^ Although false positives are uncommon (0.2%–2.4%), a relatively high rate of false negatives represents another drawback of FNAC in clinical practice.^10,45–47^ For this reason, the low rate of false negatives in our study (1.3%) compared with others was particularly encouraging.^34,36,37,39,42,45,47,48^

The lack of false positives and, especially, the low proportion of false negatives is justified by the systematic approach performed by experienced cytopathologists and further examination of specimens by a specialized immunophenotyping unit, reflecting an enhanced quality of FNAC diagnostic samples. The presence of both the cytopathologist and the patient in a separate, sufficiently equipped cytology room (intended for FNAC studies exclusively) where samples are carefully checked and further sampling performed as necessary helps also to retrieve optimal specimens.

In our experience, a skilled cytopathologist performing an FNAC of a clinically likely malignant lymphadenopathy should be able, in most cases, to quickly differentiate a metastatic tumor from lymphoma. Based on our results, patients with unexplained peripheral lymphadenopathy whose characteristics make the clinician suspect malignancy can safely undergo a rapid FNAC first. If solid malignancy is confirmed, the patient should be immediately referred to the oncologist, who may then complete the staging of disease and start treatment, although, in some cases, he or she may order an additional histological study for molecular diagnosis. If lymphoma is reported by FNAC, even though a biopsy will be necessary in most cases,^45^ the clinician may immediately refer the patient to the hematologist while simultaneously ordering an excisional biopsy. The hematologist may then have the biopsy report earlier than if it is he or she who first orders the procedure, and may complete the staging and start treatment.

Perhaps the most important study limitation is that ours was a single-institution study. Yet the unit attended patients from 12 PHC centers and the sample evaluated is representative of other QDU populations in Spain.^6–9^ In addition, owing to the relatively small sample (n = 81) and short time elapsed since the creation of our QDU (2005) or the onset of the study (2006), it is premature to assess any survival benefit (eg, improvement in 5-years overall survival) of the model in patients with lymphoma as a result of shortened waiting times. Of note, however, even though it may be intuitively expected that earlier diagnosis leads to improved outcomes, the impact of delayed presentation and diagnosis of lymphoma on survival is uncertain.^27,32,33,49,50^

## CONCLUSION

A distinct internal medicine outpatient quick diagnostic clinic allows an expeditious, agile, and prearranged system to diagnose unexplained peripheral lymphadenopathy, most notably malignant lymphadenopathy. Because of the close collaboration with the cytopathology unit and the FNAC methodical approach, the diagnostic and treatment waiting times of patients with malignancy fulfilled national and international time frame standards. In the case of lymphoma, diagnostic waiting times were shorter than those reported in studies using conventional diagnostic pathways. The reliability and accuracy of FNAC may be high provided that the samples are handled by expert cytopathologists and further tested by someone skilled in immunocytochemistry and flow cytometry. This particular diagnostic delivery unit could help overcome the difficulties facing PHC, ED, and other physicians when trying to provide rapid access to diagnostic tests to patients with troublesome lymphadenopathy.
